# Genetic analysis of tomato brown rugose fruit virus reveals evolutionary adaptation and codon usage bias patterns

**DOI:** 10.1038/s41598-024-72298-y

**Published:** 2024-09-11

**Authors:** Abozar Ghorbani

**Affiliations:** Nuclear Agriculture Research School, Nuclear Science and Technology Research Institute (NSTRI), Karaj, Iran

**Keywords:** Tomato brown rugose fruit virus, Codon usage bias, Selection pressures, Evolutionary dynamics, Molecular adaptation, Biotechnology, Evolution

## Abstract

Tomato brown rugose fruit virus (ToBRFV) poses a significant threat to tomato production worldwide, prompting extensive research into its genetic diversity, evolutionary dynamics, and adaptive strategies. In this study, we conducted a comprehensive analysis of ToBRFV at the codon level, focusing on codon usage bias, selection pressures, and evolutionary patterns across multiple genes. Our analysis revealed distinct patterns of codon usage bias and selection pressures within the ToBRFV genome, with varying levels of genetic diversity and evolutionary constraints among different genes. We observed a transition/transversion bias of 2.07 across the entire ToBRFV genome, with the movement protein (MP) gene exhibiting the highest transition/transversion bias and SNP density, suggesting potential evolutionary pressures or a higher mutation rate in this gene. Furthermore, our study identified episodic positive selection primarily in the MP gene, highlighting specific codons subject to adaptive changes in response to host immune pressures or environmental factors. Comparative analysis of codon usage bias in the coat protein (CP) and RNA-dependent RNA polymerase (RdRp) genes revealed gene-specific patterns reflecting functional constraints and adaptation to the host's translational machinery. Our findings provide valuable insights into the molecular mechanisms driving ToBRFV evolution and adaptation, with implications for understanding viral pathogenesis, host-virus interactions, and the development of control strategies. Future research directions include further elucidating the functional significance of codon usage biases, exploring the role of episodic positive selection in viral adaptation, and leveraging these insights to inform the development of effective antiviral strategies and crop protection measures.

## Introduction

The agricultural sector is a crucial pillar for global food security, providing sustenance for the world's burgeoning population. Among the myriad challenges faced by the sector, plant diseases caused by viruses are particularly pernicious, posing a grave threat to crop yields and quality. In the realm of viral plant pathogens, the Tomato brown rugose fruit virus (ToBRFV) has emerged as a particularly troublesome entity due to its adverse impact on tomato crops around the globe^1–3^. The virus, a member of the Tobamovirus genus, has been responsible for significant fruit losses, leading to economic hardship for farmers and stakeholders within the agricultural supply chain^4^. The battle against ToBRFV is a testament to the ongoing struggle to maintain crop health and productivity in the face of evolving plant diseases.

The genetic diversity of ToBRFV and its mechanisms of resistance is of paramount importance in the formulation of effective management strategies aimed at mitigating the virus's impact on tomato production^5,6^. This introduction serves to illuminate the critical role that genetic analysis plays in deciphering the virus's modus operandi and in the development of resistant tomato cultivars. A robust understanding of the genetic underpinnings of ToBRFV is essential for tackling the challenges posed by this virus^7^.

The genome of ToBRFV consists of a single-stranded RNA molecule encoding four open reading frames (ORFs), including the coat protein (CP), movement protein (MP), RNA-dependent RNA polymerase (RdRp), and a protein of unknown function. The CP, MP, and RdRp genes play crucial roles in viral replication, movement, and pathogenicity. Consequently, analyzing these genes can provide insights into the genetic variability and evolutionary relationships among different ToBRFV isolates^2,6^.

Given the virus's capacity to evolve rapidly and adapt to different environments, it is vital to investigate the genetic diversity within the ToBRFV population. The study of nucleotide substitutions in the ToBRFV movement protein gene, particularly those associated with the breaking of resistance in wild tomato species such as *Solanum habrochaites* and *Solanum peruvianum*, underscores the virus's ability to circumvent the genetic defenses of its hosts^8^. The emergence of such resistance-breaking strains necessitates constant vigilance and an adaptable approach to resistance breeding.

In this study, we conducted a comprehensive analysis of 215 whole-genome sequences of ToBRFV obtained from the National Center for Biotechnology Information (NCBI). Our analysis focused on elucidating the phylogenetic relationships among these isolates, assessing the genetic diversity within the viral population, and characterizing the molecular evolution of key genomic regions. Our study provides a comprehensive analysis of the genomic diversity, evolutionary dynamics, and molecular characteristics of ToBRFV. These findings contribute to our understanding of viral evolution and adaptation, with implications for disease management strategies and breeding programs aimed at enhancing tomato resistance to ToBRFV infection.

## Materials and methods

### Sample collection and genome sequencing

A total of 215 whole-genome sequences of ToBRFV were obtained from the National Center for Biotechnology Information (NCBI) database from Asia, Europe, Africa and America (Supplementary data 1). These sequences were derived from diverse geographical locations and represented a broad spectrum of ToBRFV isolates. Each sequence was carefully curated to ensure high-quality data for downstream analysis. We filtered unverified sequences in NCBI and sequences with gaps and ambiguous nucleotides.

### Sequence alignment and phylogenetic analysis

The amino acid sequences of three key genes MP, CP and RdRp were extracted from each ToBRFV genome sequence. These sequences were concatenated to create a composite sequence representing the entire coding region. Multiple sequence alignment (MSA) was performed using MUSCLE to align the concatenated sequences, ensuring accurate positioning of homologous amino acids.

Phylogenetic analysis was conducted using the maximum likelihood (ML) method implemented in MEGA 11^9^. The best-fitting substitution model was selected based on the Bayesian Information Criterion (BIC), and phylogenetic trees were constructed with 1000 bootstrap replicates to assess the robustness of the inferred tree topology. Bootstrap values ≥ 60 were considered statistically significant, providing confidence in the branching patterns.

### Ancestral sequence reconstruction and evolutionary time estimation

Ancestral sequence reconstruction was performed to infer putative ancestral sequences using the ML method. This analysis aimed to identify the most likely ancestral states at each internal node of the phylogenetic tree, allowing us to trace the evolutionary history of ToBRFV. The evolutionary divergence over time was estimated using the UPGMA (Unweighted Pair Group Method with Arithmetic Mean)^10^ method, which provided insights into the temporal dynamics of ToBRFV evolution.

### Genetic variation analysis

To assess genetic variation between ToBRFV isolates, several analyses were conducted. We aligned all sequences using Clustal Omega version 1.2.2^11^. Then, the number of single nucleotide polymorphisms (SNPs) was determined for each gene and the entire genome, highlighting regions of genetic diversity using Geneious Prime 2023 with default parameters in the “Find Variation/SNPs” tool (https://www.geneious.com). Furthermore, the lengths of the CP, MP, and RdRp genes were compared to identify variations in gene size and potential functional implications.

To identify and visualize SNPs in amino acid sequences, we utilized the Biopython library (version 1.84) in Python 3 to analyze the multiple sequence alignment (MSA) data. The MSA was stored in a fasta file. The analysis was performed using a custom Python script, which first loaded the alignment data using the AlignIO module from Biopython. The script then iterated over the alignment columns to identify positions where at least one SNP was present, defined as columns containing more than one unique amino acid residue. For each SNP position identified, the script recorded the position and the number of unique amino acids observed at that position. This information was used to generate a bar plot using the Matplotlib library (version 3.7.2), with the x-axis representing the SNP position and the y-axis representing the number of unique amino acids (i.e., SNP frequency) at each position. The plot was designed to facilitate easy interpretation of the SNP distribution across the amino acid sequence. The resulting plot was saved as a PDF file with a resolution of 300 DPI for inclusion in this manuscript. The Python code used for this analysis is in Supplementary data 2.

### Evolutionary divergence analysis

Average evolutionary divergence over sequence pairs within and between groups was calculated using MEGA 11. This analysis provided quantitative measures of genetic distance, allowing us to assess the degree of divergence among ToBRFV isolates. By comparing divergence levels within and between groups, we gained insights into the genetic relationships and population structure of ToBRFV.

The number of base substitutions per site from averaging over all sequence pairs within each group and between groups is shown. Analyses were conducted using the Maximum Composite Likelihood model. This analysis involved 215 nucleotide sequences. All ambiguous positions were removed for each sequence pair (pairwise deletion option). There was a total of 6395 positions in the final dataset.

The estimated Transition/Transversion bias (R) and substitution pattern and rates were estimated under the Kimura (1980) 2-parameter model. For estimating ML values, a tree topology was automatically computed. The maximum Log-likelihood for this computation was − 19,082.264. This analysis involved 215 nucleotide sequences. There was a total of 6395 positions in the final dataset.

### Genetic distance matrix

The genetic distances between ToBRFV sequences from various geographic locations were calculated and organized into a matrix using MEGA 11. This matrix served as the input data for the Principal Component Analysis (PCA). To explore the independence of genetic distances from geographic distances, PCA was performed on the genetic distance matrix using a code in Python 3 (Supplementary data 2). The genetic distance matrix was organized as a nested list, where each row and column corresponded to a country, and each value represented the genetic distance between the respective countries. The countries included in the analysis were Jordan, Peru, Israel and the State of Palestine, Germany, Mexico, Italy, United Kingdom, Canada, Greece, Netherlands, Egypt, USA, China, Turkey, and Belgium. The nested list was converted into a Panda DataFrame for ease of manipulation and analysis. DataFrame had countries as both row and column labels. PCA was performed using the PCA class from the sklearn.decomposition module in Python. The analysis was conducted to reduce the dimensionality of the data to two principal components, which explained the most variance in the genetic distances. The results of the PCA were visualized using a scatter plot, where each point represented a country. The countries were color-coded for clarity, and the first two principal components were plotted to illustrate the genetic relationships.

### Selective pressure analysis

To investigate selective pressures acting on ToBRFV genes, the ratio of nonsynonymous to synonymous substitutions (dN/dS) was calculated for the CP, MP, and RdRp genes. This analysis, performed using the Datamonkey web server (https://www.datamonkey.org/meme/)^12^, assessed the relative contributions of positive selection, purifying selection, and neutral evolution to the genetic diversity of ToBRFV.

MEME (Mixed Effects Model of Evolution) estimates a site-wise synonymous (&alpha;) and a two-category mixture of non-synonymous (&beta; with proportion p-, and &beta; + with proportion [1-p-]) rates, and uses a likelihood ratio test to determine if &beta; +  > &alpha; at a site. The estimates aggregate information over a proportion of branches at a site, so the signal is derived from episodic diversification, which is a combination of the strength of selection [effect size] and the proportion of the tree affected. A subset of branches can be selected for testing as well, in which case an additional (nuisance) parameter will be inferred from the non-synonymous rate on branches NOT selected for testing.

### Recombination analysis of ToBRFV isolates

We investigated the potential for recombination events among ToBRFV isolates. We performed a whole genome recombination analysis using GARD, a genetic algorithm for recombination detection (https://www.datamonkey.org/gard)^13^. All available complete genome sequences of ToBRFV isolates from this study were included in the analysis. Default parameters were employed for the recombination detection algorithms.

### Codon usage bias analysis

A total of 215 sequences encoding the MP, CP, and RdRp of ToBRFV were retrieved from NCBI in FASTA format. These sequences underwent preprocessing, including sequence trimming, quality assessment, and the removal of non-coding regions. Codon usage bias was analyzed using the R programming language (R-Studio version 9) with the coRdon package (Supplementary Data 2^14^). Codon frequencies were calculated to determine the prevalence of each codon across the dataset. Relative Synonymous Codon Usage (RSCU) values were estimated to assess the non-uniform usage of synonymous codons, while the Codon Adaptation Index (CAI) was computed to measure bias towards codons associated with highly expressed genes. The Effective Number of Codons (ENC) was calculated to evaluate overall codon usage bias. Additionally, GC content, including GC3S (GC content at synonymous third positions), was determined. Descriptive statistics, including mean, median, standard deviation, and range, were calculated for CAI, ENC, GC content, and GC3S, and their distributions were visualized using histograms. The results were then exported as CSV files for further interpretation, with a focus on their implications for gene expression and evolutionary dynamics within the ToBRFV coding regions.

## Result and discussion

### Comprehensive phylogenetic analysis of ToBRFV isolates

**Fig. 1:**
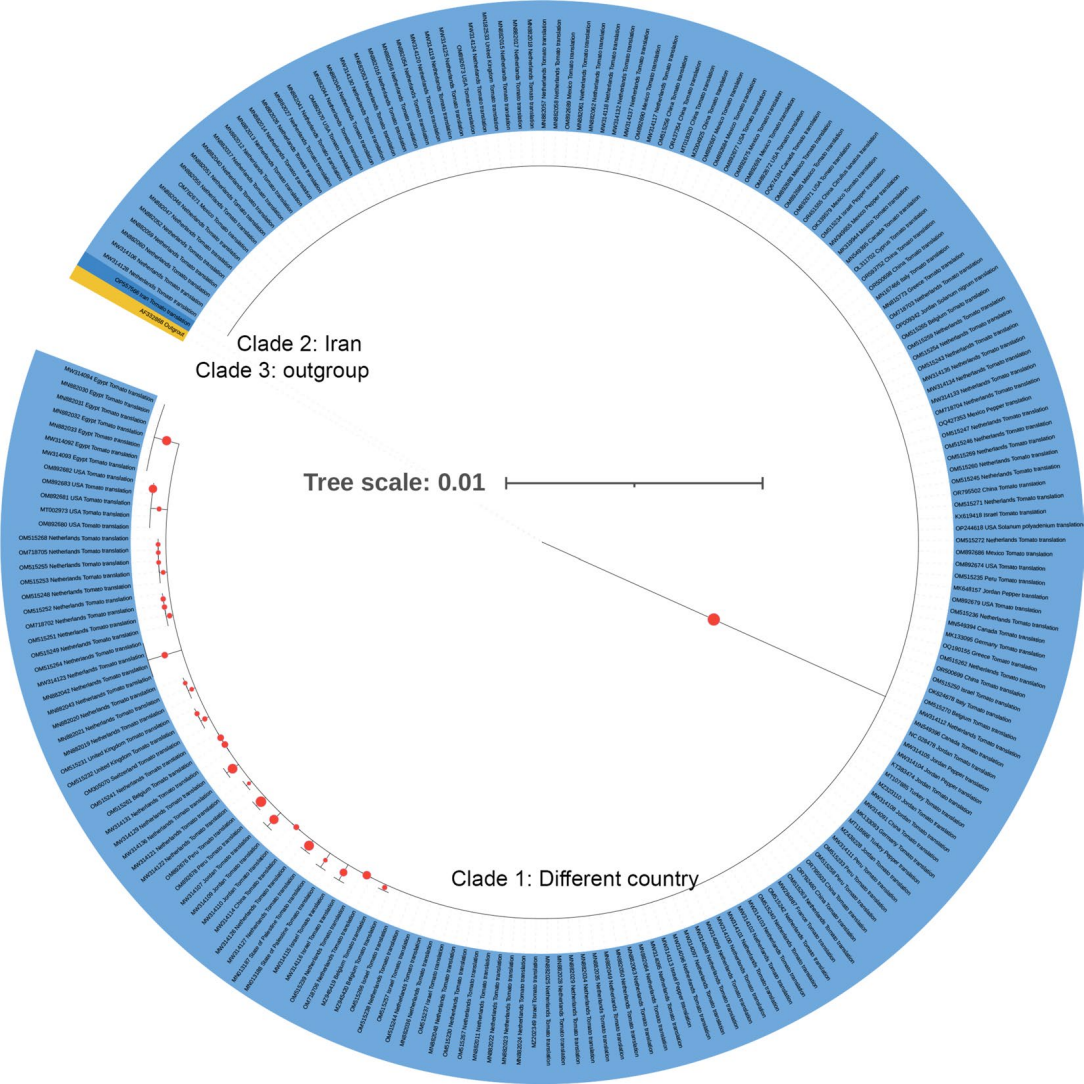
Phylogenetic relationships of ToBRFV amino acid sequences of coat protein (CP), movement protein (MP) and RNA-dependent RNA polymerase (RdRp). The tree was constructed using the maximum likelihood method and bootstrap with 1000 replications and a threshold of 60. Yellow highlight: Tomato mosaic virus amino acid sequences of CP, MP and RdRp as outgroup. Read circular show bootstrap for each node. Dark blue: Iranian isolate.

The circular phylogenetic tree representing 215 ToBRFV isolates based on their amino acid sequences revealed a complex network of evolutionary relationships. Set at a scale of 0.01, the tree allowed for a detailed analysis of genetic distances between sequences. Color-coded branches highlighted distinct phylogenetic clades, with the majority represented in blue, a specific subset (including Tomato mosaic virus amino acid sequences of CP, MP, and RdRp as outgroup) in yellow, Iranian isolates in dark blue, and red dots indicating bootstrap values for each node (Fig. 1). This visualization emphasized the remarkable genetic diversity within the ToBRFV population and provided a platform for discussing the observed evolutionary patterns and divergence in our study.

Interestingly, the phylogenetic analysis showed that isolates from different geographic locations were randomly distributed throughout the tree, without clear subgrouping based on geographical origin. This may suggest that the virus has spread globally in recent years, leading to increased genetic similarity among isolates from various regions. Our comprehensive tree construction, incorporating all coding regions of the virus (CP, MP, and RdRp), facilitated a thorough examination of the relationships between virus isolates. Notably, the Iranian isolates formed a distinct cluster, indicating the need for further investigation into their genetic characteristics and potential implications for virus spread and management.

Comparing our findings with previous studies that assessed genetic differentiation and migration patterns among ToBRFV populations from Europe, Asia, Africa and America, our results revealed differing trends. While Güller et al. reported high gene flow among geographic populations based on CP and MP gene domains, our study highlighted a lack of clear genetic differentiation between isolates from different regions. The absolute values of Fst among geographic populations being less than 0.33 and the high migration rates (> 1) observed in our study support the notion of extensive gene flow among ToBRFV populations from European, Asian, and American variants. Furthermore, the low values of Kst* and Z* metrics, and the non-significant *p*-values in pairwise comparisons for both gene regions, suggest a lack of significant genetic divergence between geographic populations. Particularly, the Snn metric results < 0 indicate minimal genetic differences between the populations, further supporting the concept of ongoing gene flow and genetic similarity among ToBRFV isolates globally^15^.

### SNP distribution analysis reveals differential evolutionary pressures on ToBRFV genes

**Fig. 2:**
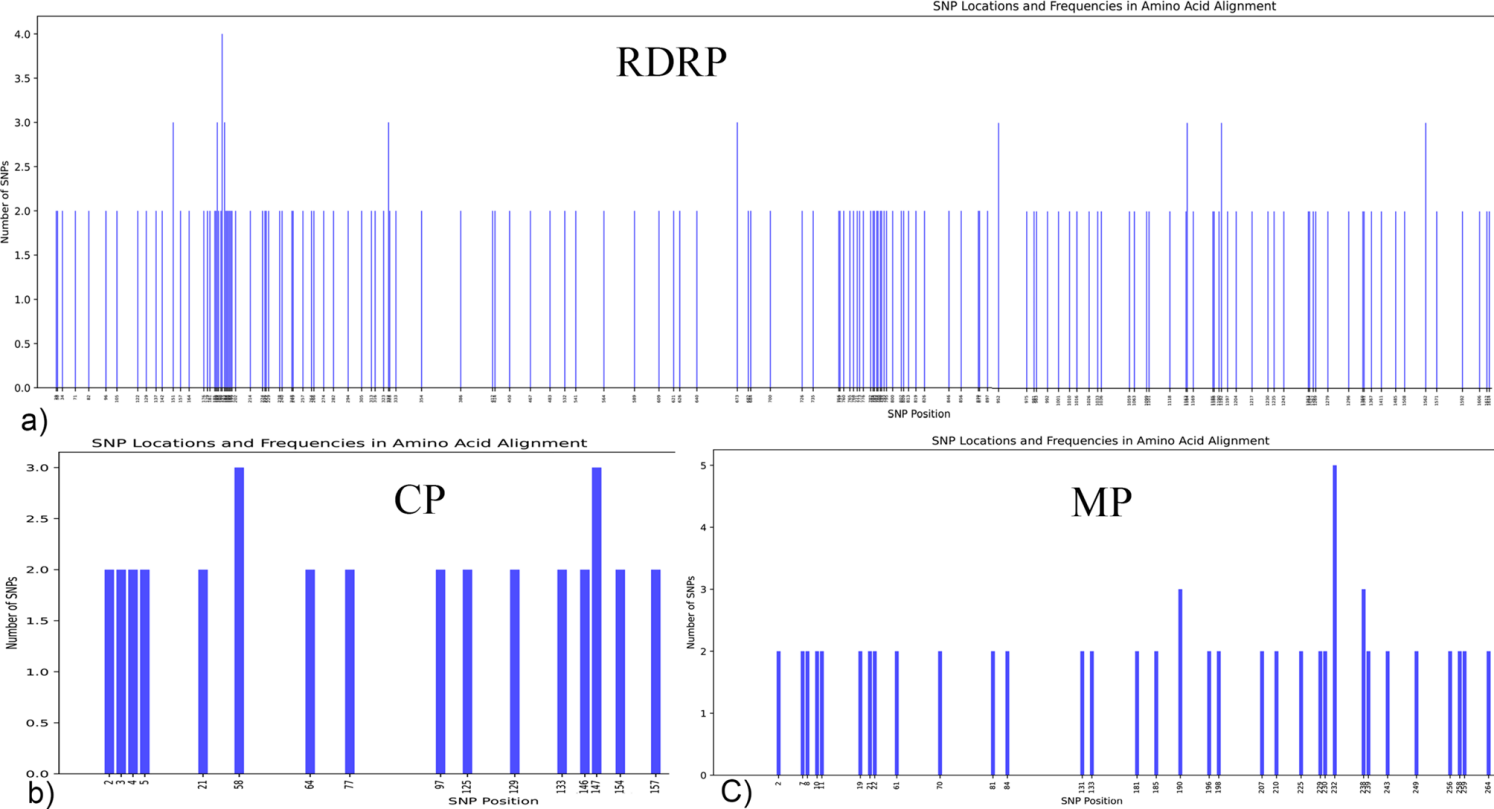
Distribution of amino acid change across ToBRFV genes: (**a**) Movement Protein (MP), (**b**) Coat Protein (CP), and c: RNA-dependent RNA polymerase (RdRp). RdRp. Amino acid change is shown in the x-axis and frequency in the y-axis color spots for each isolate. Isolates are in the left part of the plot.

The SNP distribution plot provides a visual comparison of amino acid change across the three genes of the ToBRFV: MP, CP, and RdRp. Each column represents a location of amino acid change within the respective gene sequences. The density of amino acid changes is markedly higher in the RdRp gene but if we calculate amino acid changes compared to amino acid length for each gene, we found that MP shows more variable (0.218 Number of SNP/gene length) suggesting a greater variability and potential for evolutionary adaptation (Table 2). This disparity in amino acid change distribution offers insights into the differential conservation of these genes and may have implications for the virus’s infectivity and resistance mechanisms (Fig. 2).

### Ancestral relationships of ToBRFV isolates

**Fig. 3:**
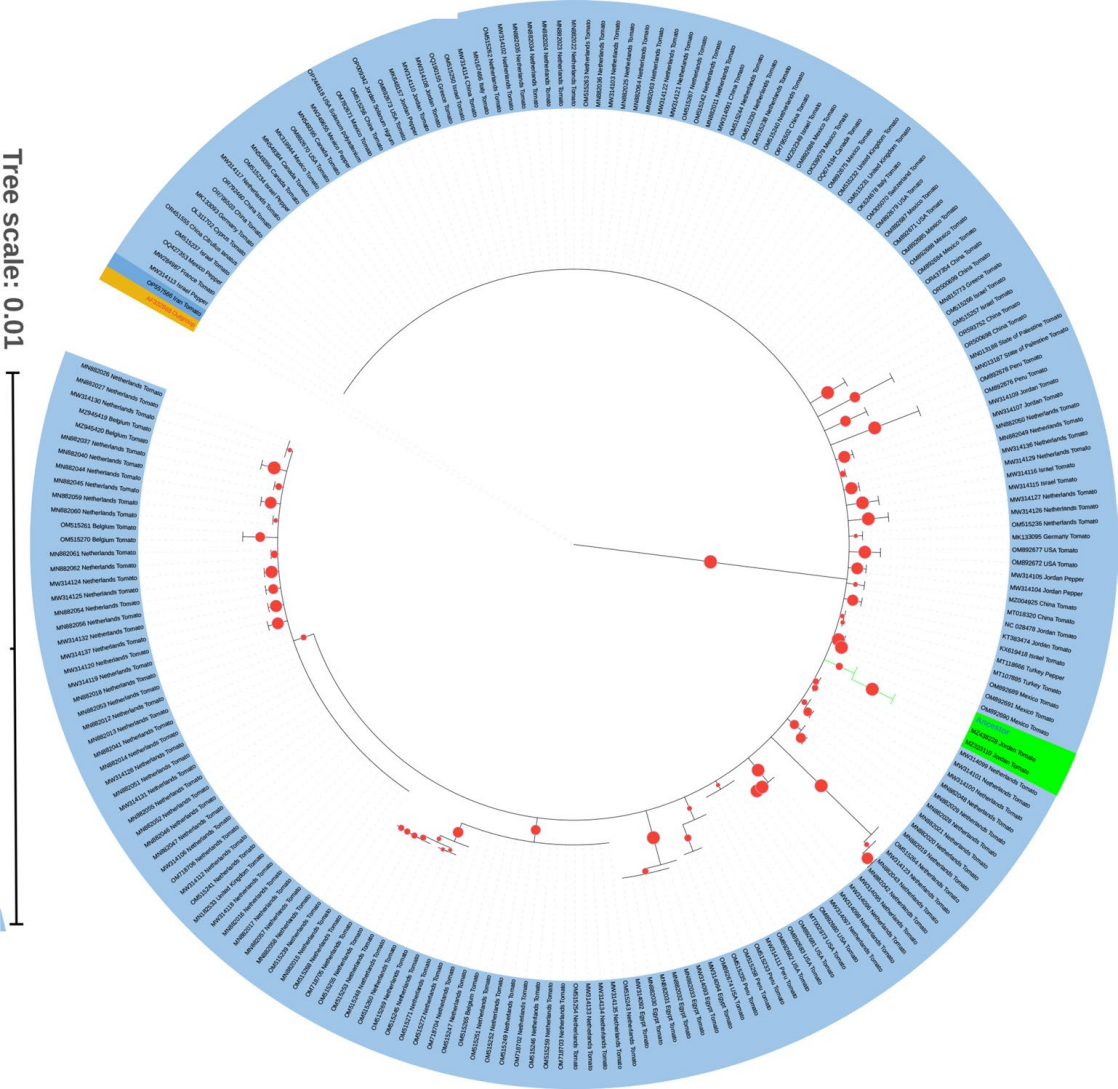
Phylogenetic tree with ancestral analysis of ToBRFV whole genome nucleotide sequences using maximum likelihood method and bootstrap with 1000 replication and threshold 60. Ancestor and sister groups were highlighted in green. Yellow highlight: Tomato mosaic virus whole genome sequences as outgroup. Read circular show bootstrap for each node. Dark blue: Iranian isolate.

Our study utilized whole genome nucleotide sequences of ToBRFV (215 isolates) to construct a circular phylogenetic tree, offering insights into the virus’s evolutionary history. The tree, with a scale set at 0.01 to reflect genetic distances between sequences, indicates significant evolutionary events. The analysis revealed two distinct groups in green representing ancestors and Jordan isolates, while the yellow section denoted TMV as the outgroup. This phylogenetic analysis not only unveiled ancestral relationships but also suggested potential evolutionary pathways of the virus, illuminating its genetic makeup and transmission dynamics (Fig. 3). Confirming the findings of Salem et al.^2^, our results supported Jordan as the origin of the virus, emphasizing the importance of investigating transmission dynamics, possibly through seed dispersal.

Interestingly, the Iranian isolate displayed a distinct clad from other isolates, signaling the presence of a potentially new strain or sequencing artifact^16^. More variation in the Iranian isolate compared to other isolates was shown in the RDRP gene where more SNP were discovered. CP and MP were more like other isolates. This observation underscores the necessity for comprehensive genomic screening to identify any novel changes that could lead to the emergence of more destructive biotypes or strains. Further investigation and sequencing of this isolate are recommended to elucidate its unique genetic characteristics and implications for viral spread and management strategies.

Moreover, insights from previous studies shed light on the emergence and evolutionary history of ToBRFV. Analysis by Yan et al. revealed a distinct clustering of tobamoviruses^17^, with ToBRFV and TMV forming sister branches, while ToMV and ToMMV grouped. Recombination events involving other tobamoviruses, as suggested by Salem et al.^2^, may have contributed to the origin of ToBRFV. The detection of a recombination event involving strains of TMV and ToMMV further underscores the complex evolutionary dynamics shaping the genetic diversity of ToBRFV^6,18^. Our analysis using the GARD algorithm did not find any evidence of recombination events among the ToBRFV isolates included in this study. A total of 2647 models were examined at a rate of 0.73 models per second. The alignment contained 638 potential breakpoints, leading to a search space of 638 models with up to 1 breakpoint. The genetic algorithm explored 414.89% of this search space.

Additionally, findings by Esmaeilzadeh et al. propose Peru as a potential center for the emergence of ToBRFV^5^, emphasizing the importance of studying isolates from diverse geographical locations. The sequencing of a ToBRFV genome from tomato seeds in Peru (MW314111) provided valuable insights into the genetic diversity of the virus, suggesting a potential origin in South America rather than the Middle East^5^. However, we used more whole genome nucleotide sequences which we believe our result (Fig. 3) is more reliable than others also, our results are confirmed by the origin of the first report of the virus^2^.

### Tomato brown rugose fruit virus diversity

The table presents the estimates of average evolutionary divergence over sequence pairs within various geographic groups. These values represent the genetic variation within each group, with a higher number indicating greater divergence (Table 1). Isolates in each country were classified. Jordan, the United Kingdom, Canada, and Mexico exhibit relatively low divergence values (0.0016 to 0.0017), suggesting a high degree of genetic similarity among the ToBRFV sequences within these populations. Peru, Israel and State of Palestine, the USA, and Belgium show moderate divergence (0.0022 to 0.0027), indicating a fair amount of genetic variation. Germany, Netherlands, and China have higher divergence values (0.0029 to 0.0034), which could reflect a more diverse set of sequences or a longer period of viral evolution within these groups. Italy and Greece stand out with the highest divergence values (0.0039 and 0.0041, respectively), pointing to significant genetic diversity within the ToBRFV sequences from these countries. Egypt shows an exceptionally low divergence value (0.0001), which might suggest a recent introduction of the virus or a very stable viral population with little genetic change. Notably, Turkey has a divergence value of 0, indicating no detectable variation among the sampled sequences, which could be due to a very recent spread or a highly conserved virus population. The genetic data reflect a bottleneck caused by eradication efforts, indicating that the virus is still undergoing geographical expansion^4^. Despite the geographic diversity, ToBRFV isolates from different regions exhibit a high level of interrelatedness, with low genetic diversity and random mutations across genomes, attributed to the introduction of infected seeds^4^.

**Table 1:** Estimates of average evolutionary divergence over sequence pairs within groups.

Jordan	0.0016
Peru	0.0026
Israel and the State of Palestine	0.0022
Germany	0.0031
Mexico	0.0017
Italy	0.0039
United Kingdom	0.0016
Canada	0.0016
Greece	0.0041
Netherlands	0.0029
Egypt	0.0001
USA	0.0023
China	0.0034
Turkey	0
Belgium	0.0027

These divergence estimates are crucial for understanding the genetic landscape of ToBRFV across different regions. They provide insights into the virus’s spread, mutation rates, and potential adaptation to diverse environmental conditions or host varieties. This information can be instrumental in developing targeted strategies for monitoring and controlling the spread of ToBRFV.

The study aimed to analyze the transition/transversion bias and density of single nucleotide polymorphisms (SNPs) across different regions of the ToBRFV genome. The obtained data revealed interesting patterns in genetic variation among ToBRFV genes (Table 2).

**Table 2:** Maximum likelihood estimates of transition/transversion bias, and number of single nucleotide polymorphisms (SNPs) in genes.

	Transition/transversion	Number of SNP/gene length
Overall	2.07	0.198
CP	2.43	0.195
MP	3.5	0.218
RDRP	2.73	0.195

Our analysis indicated a transition/transversion bias of 2.07 across the entire ToBRFV genome, with an SNP density of 0.198 per gene length. This suggests a higher frequency of transitions compared to transversions, in line with observations in RNA viruses^4^. Moreover, the CP gene exhibited a slightly higher bias of 2.43, indicating a similar SNP density to the overall genome but with a preference for transitions. In contrast, the MP gene displayed a significantly higher transition/transversion bias of 3.5 and the highest SNP density at 0.218, suggesting potential evolutionary pressures or a higher mutation rate in this gene^19^.

Interestingly, the RdRp gene showed a bias of 2.73 and an SNP density of 0.195, hinting at a higher rate of transitions compared to the CP gene. These varying biases across ToBRFV genes may reflect distinct evolutionary dynamics and constraints in each gene, underscoring the importance of considering gene-specific factors in mutational processes^20^.

Comparing our findings with previous studies, we observed a high level of genetic similarity among ToBRFV sequences, with up to 43 SNPs identified^7^. The CP gene emerged as the most conserved region, displaying low genetic variation and high conservation levels, possibly linked to elicitor recognition mechanisms in host plants^21^.

In contrast, the MP gene exhibited the highest nucleotide diversity, consistent with its role in overcoming plant resistance mechanisms such as the Tm-22 gene. Notably, specific amino acids in the MP gene have been identified as critical for evading host resistance, emphasizing the significance of genetic variation in viral adaptation^22^.

**Fig. 4:**
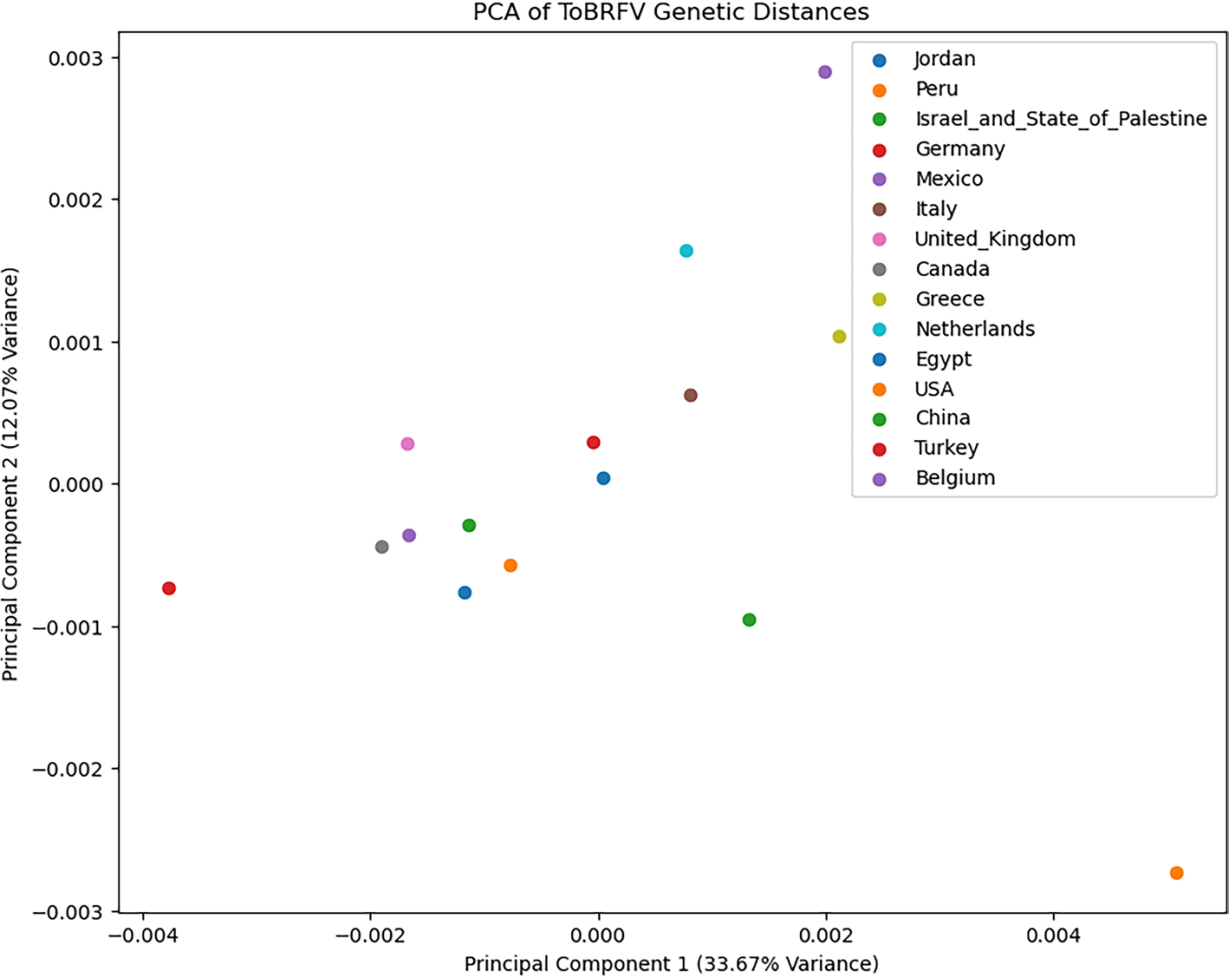
Principal component analysis (PCA) of Tomato brown rugose fruit virus (ToBRFV) genetic distances. PCA of ToBRFV genetic distances between various geographic regions. The plot visualizes the first two principal components, which capture 45.74% of the total variance in the genetic distance data. Each point represents a country, and the distance between points reflects the genetic divergence between the corresponding ToBRFV isolates.

The analysis of the average evolutionary divergence between geographic groups of ToBRFV isolates revealed insightful observations regarding the genetic relationships and variability among different populations. Our findings indicate distinct patterns of divergence within various regions, shedding light on the evolutionary dynamics of ToBRFV. Notably, the PCA of the genetic distance data, as illustrated in the provided plot (Fig. 4), reveals significant differences among geographic groups. The PCA scatter plot shows the distribution of countries based on the first two principal components, with PC1 (33.67% variance) and PC2 (12.07% variance) together capturing 45.74% of the total variance in the genetic distances. Countries such as Jordan and Turkey are positioned close to each other, indicating low genetic distances and suggesting a close genetic relationship or recent common ancestry. In contrast, Peru, Germany, Italy, Greece, and Belgium are spread out further along the principal components, displaying higher genetic distances. This suggests a greater degree of genetic variation, possibly due to prolonged separate evolution or adaptation to diverse environments. Israel and the State of Palestine, Mexico, the United Kingdom, Canada, the Netherlands, Egypt, and the USA exhibit moderate divergence. These countries balance genetic similarity and diversity, reflecting intermediate positions on the PCA plot. China, positioned distinctly on the PCA plot, shows moderate to high divergence, suggesting a unique evolutionary path or a diverse set of isolates within the region.

Our results align with previous research indicating low gene flow and limited genetic variability in ToBRFV populations. The high nucleotide identities among isolates from different regions imply a common origin, possibly linked to contamination through infected seeds or the exchange of infected fruit between countries^5^.

Furthermore, Abrahamian et al.^4^ observed that ToBRFV diverges from neutral evolutionary theory, indicating the virus is not undergoing natural selection and that accumulated mutations are low-frequency and random. The virus appears to not undergo natural selection, with accumulated mutations being low-frequency and random. The divergence from neutrality is most likely caused by a population expansion of ToBRFV, supported by the absence of any structuring in the phylogenetic tree. These insights are crucial for ensuring the continued efficacy of current diagnostic tools^4^.

### Codon-level analysis reveals episodic positive selection in the ToBRFV MP gene

**Fig. 5:**
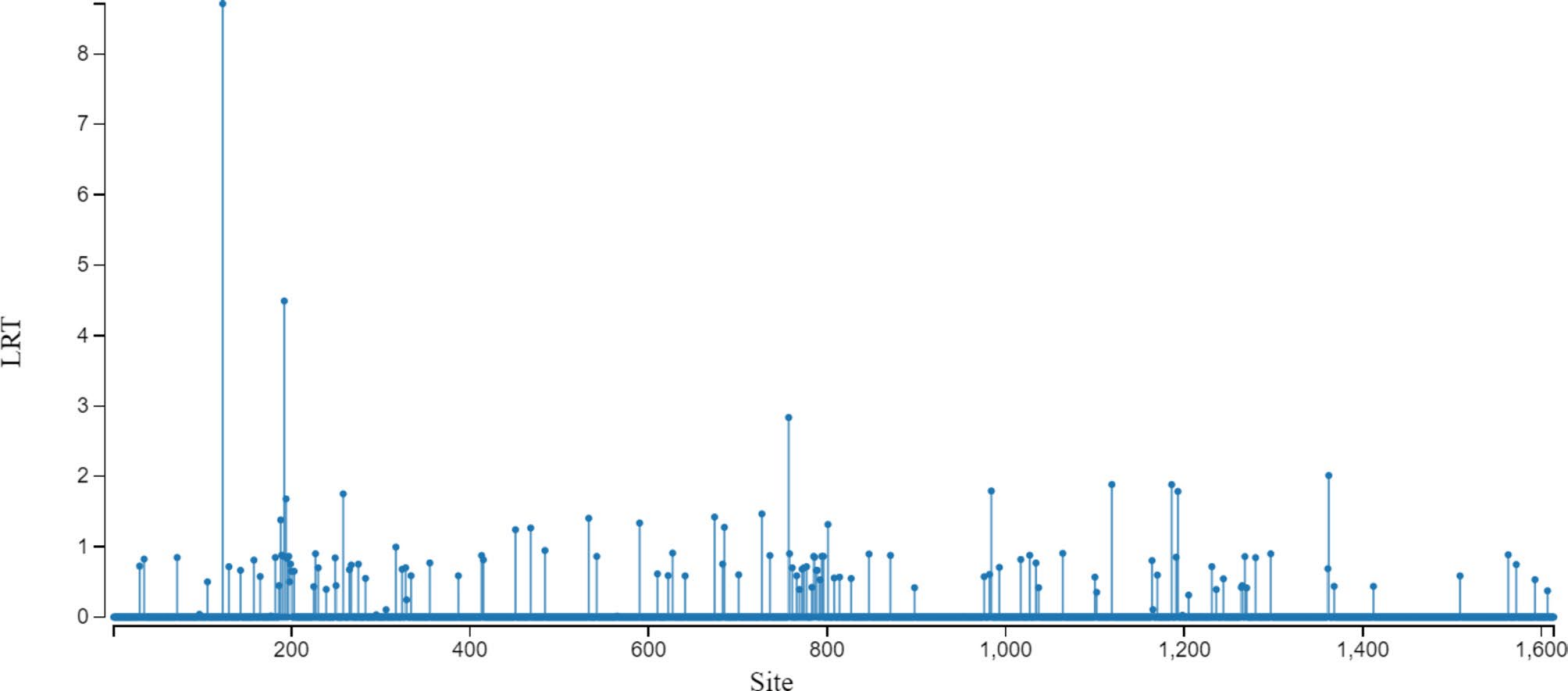
Detection of positive selection in movement protein (MP) of Tomato brown rugose fruit virus (ToBRFV) using dn/ds ratios. The figure illustrates the results from the Mixed Effects Model of Evolution (MEME) analysis, highlighting sites under positive selection in the ToBRFV genome. The x-axis enumerates the genomic sites, while the y-axis displays the Likelihood Ratio Test (LRT) values, which are indicative of positive selection when elevated. Peaks in the LRT values, represented by vertical bars, pinpoint the locations where the non-synonymous to synonymous substitution ratio (dN/dS) exceeds one, suggesting adaptive evolutionary changes.

Our study conducted a selection analysis on specific codons within the ToBRFV genome to investigate episodic positive selection and evolutionary dynamics. Utilizing a likelihood ratio test (LRT), we identified episodic positive selection primarily in the MP gene, with significant findings at codons 123 and 192 (Fig. 5). These findings suggest that certain codons within the ToBRFV genome are subject to episodic positive selection, which may be indicative of adaptive changes in response to host immune pressures or other environmental factors. The detection of these sites is crucial for understanding the evolutionary dynamics of the virus and could have implications for antiviral strategies.

For codon 123 (GTt > ACt), belonging to the first set of codons analyzed, we observed a non-synonymous rate (beta) of 1568.25 with a weight of 1.00, indicating strong positive selection. The LRT value of 8.712 (*p* = 0.0057) confirmed episodic selection at this site. Similarly, codon 192 exhibited a significant non-synonymous rate of 228.83, with an LRT value of 4.490 (*p* = 0.0491), signifying episodic positive selection (Table 3).

**Table 3:** Evidence of episodic selection in movement protein (MP) of Tomato brown rugose fruit virus (ToBRFV) genes at specific codon sites.

Codon	Partition	Alpha	Non-syn rate (beta) distribution, rates: weights	LRT	Episodic selection detected?
123 GTt > ACt	1	0.000	0.00/1568.25:0.00/1.00	8.712	Yes, *p* = 0.0057
192 Gaa > Aaa gAa > gGa Gaa > Caa	1	0.000	0.00/228.83:0.00/1.00	4.490	Yes, *p* = 0.0491

All three genes were analyzed and the positive selection was just in the MP gene.

These findings suggest that certain codons in the ToBRFV genome undergo adaptive changes in response to environmental factors or host pressures, which are crucial for understanding the virus’s evolutionary dynamics and have implications for vaccine design and antiviral strategies (Table 3).

Comparing our results to previous studies, Güller et al. and Esmaeilzadeh et al. reported strong purifying (negative) selection on the MP and CP gene domains of ToBRFV. Our study corroborates these findings, indicating that negative selection is the predominant force shaping the evolution of the ToBRFV genome. Additionally, Çelik et al. highlighted strong negative evolutionary constraints on the ORFs of ToBRFV^5,15,20^ However, we found two coding regions of MP under positive selective pressures, suggesting potential adaptive changes in these regions.

Furthermore, our study aligns with the findings of Hak and Spiegelman and Yan et al. regarding specific residues in the ToBRFV MP gene involved in evading host resistance mechanisms. Hak and Spiegelman identified residues in the central region of MP critical for escaping recognition, while Yan et al. demonstrated the importance of residues H67, N125, K129, A134, I147, and I168 in evading Tm-22-mediated resistance^19,22^. Our identification of coding positions 123 and 192 in the central region of MP confirms the previous studies and suggests that these residues are also important in resistance breaking. This indicates that the evolutionary pressures on the MP gene may be driving adaptations that enhance the virus's ability to overcome host defenses. Our findings support the notion that specific residues in the MP gene are under positive selection and play a crucial role in resistance breaking. The identification of these adaptive changes highlights the importance of further investigating these coding regions to understand their role in the virus's ability to evade host resistance mechanisms. Future studies should focus on the functional implications of these residues to develop strategies for managing ToBRFV infections.

### Codon usage bias analysis of tomato brown rugose fruit virus genome analysis

**Fig. 6:**
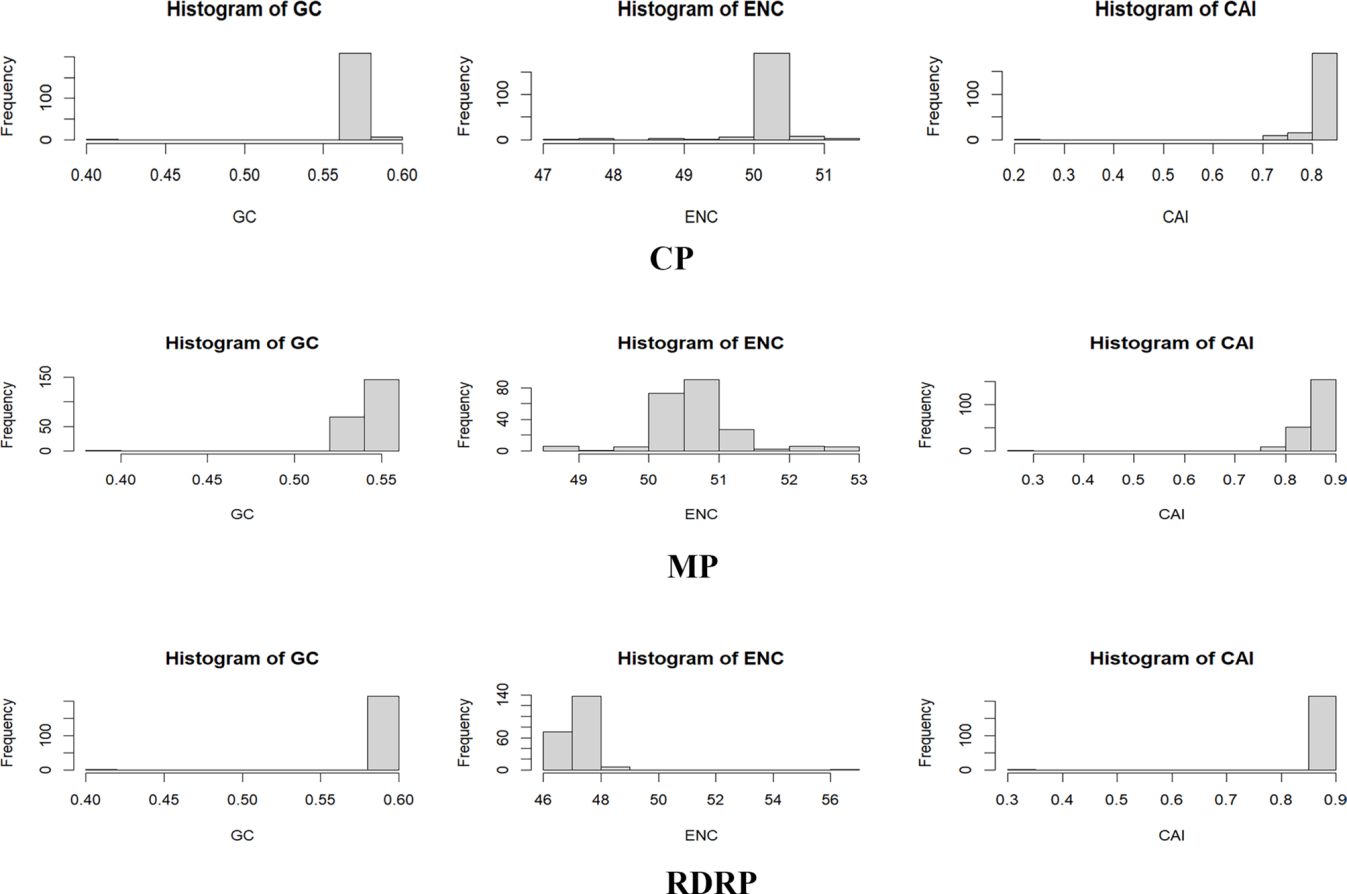
Codon usage bias analysis for Tomato Brown Rugose Fruit Virus (ToBRFV) genes: Movement Protein (MP), Coat Protein (CP), and RNA-dependent RNA Polymerase (RdRp). The figure displays histograms representing the codon usage bias for the MP, CP, and RdRp genes of the ToBRFV. The histograms compare the GC content, Effective Number of Codons (ENC), and Codon Adaptation Index (CAI) across these genes.

The comparative analysis of the codon usage bias in the MP, CP, and RdRp genes of ToBRFV revealed distinct patterns of codon usage bias and adaptation to the host’s translational machinery (Fig. 6, Supplementary Table 1–3). The codon usage patterns observed in our study align with previous research findings in plant viruses^23,24^. Our results indicate that there are significant differences in codon preferences among the genes, reflecting the functional importance and evolutionary pressures experienced by each gene.

In our study, the GC content in the CP gene was centered around 55%, suggesting a moderate GC bias that could influence codon usage and protein structure (Fig. 6). The Effective Number of Codons (ENC) values for CP indicated a moderate level of codon bias, reflecting the balance between mutational pressure and natural selection, as similarly observed in various plant RNA viruses^25^. The Codon Adaptation Index (CAI) peaks in CP at around 0.7, indicating a moderate level of adaptation to the host’s translational machinery^26^. In contrast, Gómez et al. reported that PVY genes had lower CAI values compared to their hosts, suggesting that PVY is less adapted to its hosts than ToBRFV, which highlights the variability in codon adaptation among different plant viruses^27^.

For the MP gene, the GC content peaked at about 50%, slightly lower than CP, potentially impacting the amino acid composition of the protein. The ENC distribution for MP suggested a similar level of codon bias as CP, while the CAI peaks at around 0.8 indicated a higher adaptation to the host’s translational efficiency compared to CP^26^.

In the RdRp gene, the GC content peaked slightly above MP, hinting at a potential for a more stable RNA structure. The broader distribution of ENC values for RdRp suggested less codon usage bias, indicating a more diverse set of codons used for encoding amino acids. The CAI for RdRp, although similar to MP, was slightly lower, suggesting a lesser degree of adaptation to the host’s translational machinery compared to MP^26^.

Our findings support the idea that codon usage patterns are influenced by a combination of factors, including mutational bias and translational selection, rather than solely translational selection^23,24,28^. He et al. highlighted that these factors, along with gene length, secondary protein structure, and selective transcription, play significant roles in shaping codon usage bias in plant RNA viruses^25^. This is corroborated by the ENC–GC3S plot analysis in PVY, which showed clustering below the expected curve, indicating the influence of GC content on codon usage^27^. The observed differences in codon preferences among genes within the same genome may be attributed to varying evolutionary pressures and functional constraints, similar to the findings in Potato Virus M^28^. While Cheeran et al. demonstrated that natural selection predominantly shapes codon usage bias in TMV genes, our analysis of ToBRFV suggests a nuanced interplay between mutational bias and translational selection, reflecting unique evolutionary trajectories in different viral genomes^29^.

Understanding these patterns of codon usage bias and adaptation to the host’s translational machinery in ToBRFV genes can provide valuable insights into the virus’s evolution and host-virus interactions. He et al. demonstrated that host selection pressure significantly influences the codon usage patterns of plant RNA viruses, suggesting that ToBRFV may similarly evolve to optimize its replication and survival in its host^25^. This is further supported by the work of Gómez et al., who demonstrated that PVY strains also show similar codon usage preferences, underscoring shared evolutionary mechanisms among plant-infecting viruses^27^.

The RSCU values for the CP gene of ToBRFV were rigorously analyzed, uncovering significant codon bias patterns. These patterns closely resemble those found in potato virus Y (PVY) strains, which also exhibit a preference for codons ending in A or U^27^. RSCU serves as a metric for comparing the observed frequency of codons to the expected frequency under equal usage of synonymous codons. Notably, codons associated with Phenylalanine (Phe), Leucine (Leu), Serine (Ser), and Proline (Pro) exhibited varying degrees of bias towards specific codons, as evidenced by their RSCU values. For instance, the Proline codons CCC and CCG demonstrated substantial bias with RSCU values of 1.00464 and 2.450116, respectively, indicating a distinct preference for these codons. Similarly, Histidine (His) and Glutamine (Gln) codons displayed a pronounced bias towards CAC and CAA, respectively, with RSCU values approximating 2, suggestive of their preferential usage within the CP gene. Moreover, the Arginine (Arg) codons, particularly CGC and AGA, manifested noticeable bias, underscored by their elevated RSCU values, indicative of a predilection for these codons within the CP gene of ToBRFV (Table 4).

**Table 4:** Relative synonymous codon usage (RSCU) analysis for the coat protein (CP) gene of Tomato brown rugose fruit virus (ToBRFV).

	Aa_code	Amino_acid	Codon	Subfam	Cts	Rscu	W_cai	RSCU
1	F	Phe	TTT	Phe_TT	6	0.7	1	1.4
2	F	Phe	TTC	Phe_TT	2	0.3	0.428571	0.6
3	L	Leu	TTA	Leu_TT	6	0.583333	1	1.166667
4	L	Leu	TTG	Leu_TT	4	0.416667	0.714286	0.833333
5	S	Ser	TCT	Ser_TC	3	0.307692	1	1.230769
6	S	Ser	TCC	Ser_TC	2	0.230769	0.75	0.923077
7	S	Ser	TCA	Ser_TC	3	0.307692	1	1.230769
8	S	Ser	TCG	Ser_TC	1	0.153846	0.5	0.615385
9	Y	Tyr	TAT	Tyr_TA	1	0.333333	0.5	0.666667
10	Y	Tyr	TAC	Tyr_TA	3	0.666667	1	1.333333
11	C	Cys	TGT	Cys_TG	1	0.666667	1	1.333333
12	C	Cys	TGC	Cys_TG	0	0.333333	0.5	0.666667
13	W	Trp	TGG	Trp_TG	3	1	1	1
14	L	Leu	CTT	Leu_CT	0	0.142857	0.25	0.571429
15	L	Leu	CTC	Leu_CT	0	0.142857	0.25	0.571429
16	L	Leu	CTA	Leu_CT	3	0.571429	1	2.285714
17	L	Leu	CTG	Leu_CT	0	0.142857	0.25	0.571429
18	P	Pro	CCT	Pro_CC	6	0.00406	0.006629	0.016241
19	P	Pro	CCC	Pro_CC	432	0.25116	0.410038	1.00464
20	P	Pro	CCA	Pro_CC	227	0.132251	0.215909	0.529002
21	P	Pro	CCG	Pro_CC	1055	0.612529	1	2.450116
22	H	His	CAT	His_CA	0	0.002179	0.002183	0.004357
23	H	His	CAC	His_CA	457	0.997821	1	1.995643
24	Q	Gln	CAA	Gln_CA	1311	0.654691	1	1.309381
25	Q	Gln	CAG	Gln_CA	691	0.345309	0.527439	0.690619
26	R	Arg	CGT	Arg_CG	1	0.000864	0.001601	0.003454
27	R	Arg	CGC	Arg_CG	1248	0.539292	1	2.157168
28	R	Arg	CGA	Arg_CG	840	0.363126	0.673339	1.452504
29	R	Arg	CGG	Arg_CG	223	0.096718	0.179343	0.386874
30	I	Ile	ATT	Ile_AT	1	0.2	0.333333	0.6
31	I	Ile	ATC	Ile_AT	1	0.2	0.333333	0.6
32	I	Ile	ATA	Ile_AT	5	0.6	1	1.8
33	M	Met	ATG	Met_AT	1	1	1	1
34	T	Thr	ACT	Thr_AC	6	0.002291	0.00413	0.009165
35	T	Thr	ACC	Thr_AC	884	0.289689	0.522124	1.158756
36	T	Thr	ACA	Thr_AC	1694	0.554828	1	2.219313
37	T	Thr	ACG	Thr_AC	467	0.153191	0.276106	0.612766
38	N	Asn	AAT	Asn_AA	9	0.00579	0.005824	0.011581
39	N	Asn	AAC	Asn_AA	1716	0.99421	1	1.988419
40	K	Lys	AAA	Lys_AA	2110	0.562184	1	1.124368
41	K	Lys	AAG	Lys_AA	1643	0.437816	0.778778	0.875632
42	S	Ser	AGT	Ser_AG	2	0.010309	0.010417	0.020619
43	S	Ser	AGC	Ser_AG	287	0.989691	1	1.979381
44	R	Arg	AGA	Arg_AG	1440	0.805478	1	1.610956
45	R	Arg	AGG	Arg_AG	347	0.194522	0.241499	0.389044
46	V	Val	GTT	Val_GT	3	0.210526	0.8	0.842105
47	V	Val	GTC	Val_GT	4	0.263158	1	1.052632
48	V	Val	GTA	Val_GT	4	0.263158	1	1.052632
49	V	Val	GTG	Val_GT	4	0.263158	1	1.052632
50	A	Ala	GCT	Ala_GC	6	0.003091	0.00791	0.012362
51	A	Ala	GCC	Ala_GC	562	0.248565	0.636158	0.99426
52	A	Ala	GCA	Ala_GC	884	0.390728	1	1.562914
53	A	Ala	GCG	Ala_GC	809	0.357616	0.915254	1.430464
54	D	Asp	GAT	Asp_GA	3	0.005874	0.005908	0.011747
55	D	Asp	GAC	Asp_GA	676	0.994126	1	1.988253
56	E	Glu	GAA	Glu_GA	900	0.465152	0.869691	0.930305
57	E	Glu	GAG	Glu_GA	1035	0.534848	1	1.069695
58	G	Gly	GGT	Gly_GG	4	0.001699	0.004023	0.006796
59	G	Gly	GGC	Gly_GG	1051	0.357458	0.84634	1.429834
60	G	Gly	GGA	Gly_GG	1242	0.422358	1	1.689433
61	G	Gly	GGG	Gly_GG	642	0.218485	0.517297	0.873938

The table provides an in-depth look at the RSCU values for the CP gene of the ToBRFV. RSCU is a measure of codon bias that compares the observed frequency of codons to the expected frequency if all synonymous codons for the same amino acid were used equally. An RSCU value of 1 indicates no bias, values greater than 1 indicate a positive bias and values less than 1 indicate a negative bias.

Moreover, the analysis of the w_cai column, reflecting the relative adaptiveness of each codon, unveiled crucial insights into the codon usage patterns of ToBRFV. These findings are in line with Gómez et al., who reported that PVY strains also showed a high preference for A/U-ending codons, suggesting a common evolutionary strategy among plant viruses to optimize codon usage for host adaptation^27^. Noteworthy, values closer to 1 in the w_cai column signify higher adaptiveness, thus delineating the efficiency of gene expression and protein synthesis within ToBRFV. This comprehensive analysis not only enhances our understanding of codon usage dynamics in ToBRFV but also bears significant implications for gene expression regulation and the formulation of targeted control strategies against the virus.

Comparing our findings with those of He et al., who investigated the codon usage patterns of Narcissus late season yellows virus (NLSYV) and Narcissus yellow stripe virus (NYSV) and Narcissus degeneration virus (NDV) CP genes, a recurring preference for A/U in the third codon position across narcissus viruses was observed^30^. This comparative analysis sheds light on the shared characteristics and distinctive features of codon usage among different viruses, providing valuable insights into the evolutionary mechanisms and selective pressures governing codon bias in viral genomes. The codon usage patterns in ToBRFV show a mix of U- and G-ending codons, similar to what has been observed in Potato Virus M (He et al., 2019). This preference, despite the GC-rich or AU-rich composition, suggests the influence of mutation pressure on codon selection.

Therefore, the meticulous analysis of RSCU values and codon adaptation index in the CP gene of ToBRFV offers the essential groundwork for deciphering the intricate interplay between codon bias, gene expression, and viral evolution. These findings not only enrich our understanding of viral genome dynamics but also hold promise for informing future antiviral strategies and therapeutic interventions.

In this study, we conducted a comprehensive RSCU analysis for the MP and RdRp genes of ToBRFV (Tables 5 and 6). RSCU provides valuable insights into the preferences and biases in codon usage, shedding light on the genetic characteristics of the virus. The RSCU values, presented in Tables 5 and 6, reveal distinct patterns of codon usage in both genes, which could have significant implications for gene expression efficiency, protein synthesis, and the development of control strategies against ToBRFV.

**Table 5:** Relative synonymous codon usage (RSCU) analysis for the movement protein (MP) gene of Tomato brown rugose fruit virus (ToBRFV).

	Aa_code	Amino_acid	Codon	Subfam	Cts	Rscu	W_cai	RSCU
1	F	Phe	TTT	Phe_TT	5	0.545455	1	1.090909
2	F	Phe	TTC	Phe_TT	4	0.454545	0.833333	0.909091
3	L	Leu	TTA	Leu_TT	4	0.384615	0.625	0.769231
4	L	Leu	TTG	Leu_TT	7	0.615385	1	1.230769
5	S	Ser	TCT	Ser_TC	3	0.222222	0.5	0.888889
6	S	Ser	TCC	Ser_TC	2	0.166667	0.375	0.666667
7	S	Ser	TCA	Ser_TC	7	0.444444	1	1.777778
8	S	Ser	TCG	Ser_TC	2	0.166667	0.375	0.666667
9	Y	Tyr	TAT	Tyr_TA	4	0.5	1	1
10	Y	Tyr	TAC	Tyr_TA	4	0.5	1	1
11	C	Cys	TGT	Cys_TG	4	0.833333	1	1.666667
12	C	Cys	TGC	Cys_TG	0	0.166667	0.2	0.333333
13	W	Trp	TGG	Trp_TG	2	1	1	1
14	L	Leu	CTT	Leu_CT	5	0.375	1	1.5
15	L	Leu	CTC	Leu_CT	4	0.3125	0.833333	1.25
16	L	Leu	CTA	Leu_CT	2	0.1875	0.5	0.75
17	L	Leu	CTG	Leu_CT	1	0.125	0.333333	0.5
18	P	Pro	CCT	Pro_CC	1	0.001021	0.001828	0.004086
19	P	Pro	CCC	Pro_CC	110	0.056691	0.101463	0.226762
20	P	Pro	CCA	Pro_CC	750	0.383555	0.686472	1.534219
21	P	Pro	CCG	Pro_CC	1093	0.558733	1	2.234934
22	H	His	CAT	His_CA	2	0.005348	0.005376	0.010695
23	H	His	CAC	His_CA	557	0.994652	1	1.989305
24	Q	Gln	CAA	Gln_CA	1966	0.486159	0.946128	0.972318
25	Q	Gln	CAG	Gln_CA	2078	0.513841	1	1.027682
26	R	Arg	CGT	Arg_CG	2	0.001029	0.002086	0.004117
27	R	Arg	CGC	Arg_CG	1437	0.49331	1	1.973242
28	R	Arg	CGA	Arg_CG	788	0.270669	0.548679	1.082676
29	R	Arg	CGG	Arg_CG	684	0.234991	0.476356	0.939966
30	I	Ile	ATT	Ile_AT	8	0.5625	1	1.6875
31	I	Ile	ATC	Ile_AT	2	0.1875	0.333333	0.5625
32	I	Ile	ATA	Ile_AT	3	0.25	0.444444	0.75
33	M	Met	ATG	Met_AT	7	1	1	1
34	T	Thr	ACT	Thr_AC	3	0.001523	0.00385	0.006091
35	T	Thr	ACC	Thr_AC	1038	0.395508	1	1.582033
36	T	Thr	ACA	Thr_AC	638	0.243243	0.615014	0.972973
37	T	Thr	ACG	Thr_AC	944	0.359726	0.909528	1.438904
38	N	Asn	AAT	Asn_AA	15	0.010936	0.011057	0.021873
39	N	Asn	AAC	Asn_AA	1446	0.989064	1	1.978127
40	K	Lys	AAA	Lys_AA	4647	0.572273	1	1.144546
41	K	Lys	AAG	Lys_AA	3473	0.427727	0.747418	0.855454
42	S	Ser	AGT	Ser_AG	6	0.009576	0.009669	0.019152
43	S	Ser	AGC	Ser_AG	723	0.990424	1	1.980848
44	R	Arg	AGA	Arg_AG	2698	0.545253	1	1.090505
45	R	Arg	AGG	Arg_AG	2250	0.454747	0.834013	0.909495
46	V	Val	GTT	Val_GT	13	0.4	1	1.6
47	V	Val	GTC	Val_GT	10	0.314286	0.785714	1.257143
48	V	Val	GTA	Val_GT	4	0.142857	0.357143	0.571429
49	V	Val	GTG	Val_GT	4	0.142857	0.357143	0.571429
50	A	Ala	GCT	Ala_GC	5	0.002223	0.003859	0.008892
51	A	Ala	GCC	Ala_GC	269	0.100037	0.173633	0.400148
52	A	Ala	GCA	Ala_GC	1554	0.576139	1	2.304557
53	A	Ala	GCG	Ala_GC	867	0.321601	0.558199	1.286402
54	D	Asp	GAT	Asp_GA	11	0.013393	0.013575	0.026786
55	D	Asp	GAC	Asp_GA	883	0.986607	1	1.973214
56	E	Glu	GAA	Glu_GA	2709	0.56096	1	1.121921
57	E	Glu	GAG	Glu_GA	2120	0.43904	0.782657	0.878079
58	G	Gly	GGT	Gly_GG	11	0.002415	0.004601	0.009662
59	G	Gly	GGC	Gly_GG	969	0.19525	0.371933	0.780998
60	G	Gly	GGA	Gly_GG	2607	0.52496	1	2.099839
61	G	Gly	GGG	Gly_GG	1377	0.277375	0.528374	1.109501

The table provides a comprehensive RSCU analysis for the MP gene of the ToBRFV. RSCU is a measure that indicates the relative frequency of synonymous codons used for encoding each amino acid. An RSCU value of 1 suggests no bias, above 1 indicates a preference for that codon, and below 1 indicates avoidance.

**Table 6:** Relative synonymous codon usage (RSCU) analysis for the RNA-dependent RNA polymerase (RdRp) gene of tomato brown rugose fruit virus (ToBRFV).

	Aa_code	Amino_acid	Codon	Subfam	Cts	Rscu	W_cai	RSCU
1	F	Phe	TTT	Phe_TT	45	0.589744	1	1.179487
2	F	Phe	TTC	Phe_TT	31	0.410256	0.695652	0.820513
3	L	Leu	TTA	Leu_TT	38	0.5	1	1
4	L	Leu	TTG	Leu_TT	38	0.5	1	1
5	S	Ser	TCT	Ser_TC	36	0.377551	1	1.510204
6	S	Ser	TCC	Ser_TC	19	0.204082	0.540541	0.816327
7	S	Ser	TCA	Ser_TC	21	0.22449	0.594595	0.897959
8	S	Ser	TCG	Ser_TC	18	0.193878	0.513514	0.77551
9	Y	Tyr	TAT	Tyr_TA	34	0.514706	1	1.029412
10	Y	Tyr	TAC	Tyr_TA	32	0.485294	0.942857	0.970588
11	C	Cys	TGT	Cys_TG	24	0.757576	1	1.515152
12	C	Cys	TGC	Cys_TG	7	0.242424	0.32	0.484848
13	W	Trp	TGG	Trp_TG	14	1	1	1
14	L	Leu	CTT	Leu_CT	35	0.409091	1	1.636364
15	L	Leu	CTC	Leu_CT	16	0.193182	0.472222	0.772727
16	L	Leu	CTA	Leu_CT	16	0.193182	0.472222	0.772727
17	L	Leu	CTG	Leu_CT	17	0.204545	0.5	0.818182
18	P	Pro	CCT	Pro_CC	15	0.001005	0.00233	0.004021
19	P	Pro	CCC	Pro_CC	3854	0.242224	0.561381	0.968897
20	P	Pro	CCA	Pro_CC	6866	0.43148	1	1.725919
21	P	Pro	CCG	Pro_CC	5176	0.325291	0.753895	1.301162
22	H	His	CAT	His_CA	21	0.002775	0.002782	0.005549
23	H	His	CAC	His_CA	7906	0.997225	1	1.994451
24	Q	Gln	CAA	Gln_CA	11,534	0.52942	1	1.05884
25	Q	Gln	CAG	Gln_CA	10,252	0.47058	0.88886	0.94116
26	R	Arg	CGT	Arg_CG	8	0.000468	0.001349	0.001873
27	R	Arg	CGC	Arg_CG	6123	0.318626	0.918003	1.274506
28	R	Arg	CGA	Arg_CG	6670	0.347086	1	1.388345
29	R	Arg	CGG	Arg_CG	6415	0.333819	0.961775	1.335276
30	I	Ile	ATT	Ile_AT	37	0.431818	1	1.295455
31	I	Ile	ATC	Ile_AT	23	0.272727	0.631579	0.818182
32	I	Ile	ATA	Ile_AT	25	0.295455	0.684211	0.886364
33	M	Met	ATG	Met_AT	45	1	1	1
34	T	Thr	ACT	Thr_AC	32	0.001208	0.002819	0.004834
35	T	Thr	ACC	Thr_AC	7244	0.265317	0.618966	1.061266
36	T	Thr	ACA	Thr_AC	11,704	0.428645	1	1.714579
37	T	Thr	ACG	Thr_AC	8323	0.30483	0.711149	1.219321
38	N	Asn	AAT	Asn_AA	39	0.003893	0.003909	0.007787
39	N	Asn	AAC	Asn_AA	10,233	0.996107	1	1.992213
40	K	Lys	AAA	Lys_AA	15,986	0.535811	1	1.071622
41	K	Lys	AAG	Lys_AA	13,849	0.464189	0.866329	0.928378
42	S	Ser	AGT	Ser_AG	24	0.002665	0.002672	0.005329
43	S	Ser	AGC	Ser_AG	9356	0.997335	1	1.994671
44	R	Arg	AGA	Arg_AG	15,354	0.572905	1	1.14581
45	R	Arg	AGG	Arg_AG	11,446	0.427095	0.74549	0.85419
46	V	Val	GTT	Val_GT	41	0.311111	1	1.244444
47	V	Val	GTC	Val_GT	27	0.207407	0.666667	0.82963
48	V	Val	GTA	Val_GT	28	0.214815	0.690476	0.859259
49	V	Val	GTG	Val_GT	35	0.266667	0.857143	1.066667
50	A	Ala	GCT	Ala_GC	29	0.001415	0.002957	0.005661
51	A	Ala	GCC	Ala_GC	5457	0.257501	0.537893	1.030006
52	A	Ala	GCA	Ala_GC	10,146	0.478722	1	1.91489
53	A	Ala	GCG	Ala_GC	5560	0.262361	0.548044	1.049443
54	D	Asp	GAT	Asp_GA	67	0.006883	0.00693	0.013765
55	D	Asp	GAC	Asp_GA	9811	0.993117	1	1.986235
56	E	Glu	GAA	Glu_GA	12,496	0.526633	1	1.053266
57	E	Glu	GAG	Glu_GA	11,232	0.473367	0.898856	0.946734
58	G	Gly	GGT	Gly_GG	27	0.001094	0.002342	0.004375
59	G	Gly	GGC	Gly_GG	6962	0.271971	0.582337	1.087884
60	G	Gly	GGA	Gly_GG	11,956	0.467034	1	1.868135
61	G	Gly	GGG	Gly_GG	6653	0.259902	0.556494	1.039606

The table presents the RSCU values for the RdRp gene of ToBRFV, offering insights into codon usage preferences. RSCU values greater than 1 indicate a bias towards a particular codon, while values less than 1 suggest avoidance.

Analyzing the MP gene of ToBRFV, our results indicate specific codon preferences. Notably, Phenylalanine (Phe) codons TTT and TTC exhibit a slight preference for TTT (RSCU = 1.090909), whereas Leucine (Leu) codons favor TTG over TTA (RSCU = 1.23 and 0.76, respectively). Additionally, Serine (Ser) codons demonstrate a strong preference for TCA (RSCU = 1.777778), and Proline (Pro) codons display a significant bias towards CCG (RSCU = 2.23). Furthermore, Histidine (His), Glutamine (Gln), and Arginine (Arg) codons show notable biases towards CAC, CAG, and CGC, respectively, with RSCU values close to 2.

For the RdRp gene of ToBRFV, our analysis reveals distinct codon usage preferences. Phenylalanine (Phe) codons favor TTT over TTC, while Leucine (Leu) codons exhibit no bias between TTA and TTG. Serine (Ser) codons display a moderate bias, with TCT showing the highest RSCU value. Proline (Pro) codons show a strong bias towards CCA and CCG, and Histidine (His) and Glutamine (Gln) codons exhibit a strong bias towards CAC and CAA, respectively. Moreover, Arginine (Arg) codons display notable biases, particularly for CGA and CGC. Comparing our findings with previous studies on cucurbit-infecting tobamoviruses^26^, we observe similarities in codon usage preferences, particularly in the preference of U over C in most synonymous third codon positions. This consistency across tobamoviruses underscores the potential for targeted interventions in viral gene control and attenuation. Strategies such as deoptimizing synonymous codons less used by both the virus and host may offer avenues for reducing viral gene expression and virulence. However, considerations of codon-specific biases, such as increasing codons ending with CpA to align with host preferences, must be carefully evaluated to avoid adverse effects on viral survival.

Comparing our findings with those of Cheeran et al. on Tobacco Mosaic Virus (TMV), we observe commonalities in codon usage preferences, particularly in the prevalence of high-frequency codons ending with nucleotide T, indicative of shared evolutionary pressures shaping viral codon bias^29^. The codon usage patterns in ToBRFV show a mix of U- and G-ending codons, similar to what has been observed in Potato Virus M^28^. This preference, despite the GC-rich or AU-rich composition, suggests the influence of mutation pressure on codon selection.

Moreover, our study contributes to understanding the mutation pressures shaping codon usage in ToBRFV genes. We found a preference for A/G in TuMV protein-coding regions, indicative of mutation pressures^31^. This insight underscores the dynamic nature of codon usage and its implications for viral evolution and adaptation. The comprehensive analysis of codon usage bias in ToBRFV genes, alongside comparative studies like that of Potato Virus M^28^, provides deeper insights into the evolutionary dynamics of plant viruses. These insights are essential for developing effective antiviral strategies and improving our understanding of virus-host interactions.

In conclusion, our analysis provides valuable insights into the codon usage patterns of ToBRFV, which could inform strategies for controlling viral gene expression and virulence. Further research into the functional implications of codon biases and their interplay with host factors is warranted to deepen our understanding of virus-host interactions and aid in the development of effective control measures against ToBRFV.

## Conclusion

In conclusion, our multi-faceted analysis of ToBRFV provides valuable insights into its genetic diversity, evolutionary dynamics, and adaptive strategies. Through comprehensive phylogenetic analysis, we revealed a complex network of evolutionary relationships among ToBRFV isolates, indicating extensive global spread and significant genetic diversity. Our findings suggest ongoing gene flow among ToBRFV populations from different geographic regions, with limited genetic differentiation and notable genetic similarity observed across diverse populations. Furthermore, SNP distribution analysis unveiled differential evolutionary pressures on ToBRFV genes, emphasizing the importance of considering gene-specific factors in mutational processes. At the codon level, our study identified distinct patterns of codon usage bias and selection pressures within the ToBRFV genome. We observed varying levels of genetic diversity and evolutionary constraints among different genes, with Episodic positive selection primarily observed in the MP gene. This indicates adaptive changes in response to host immune pressures or environmental factors. Comparative analysis of codon usage bias in the CP and RdRp genes provided further insights into functional constraints and adaptation to the host's translational machinery. These findings underscore the importance of understanding codon usage dynamics in the context of viral evolution and host-virus interactions. Overall, our study enhances the understanding of ToBRFV evolution, host-virus interactions, and the molecular mechanisms driving viral adaptation. These insights can inform the development of targeted strategies for monitoring and controlling the spread of ToBRFV, as well as guide future research into viral pathogenesis and the development of antiviral interventions. Continued Collaboration and surveillance efforts are essential to stay ahead of emerging viral threats and safeguard global tomato production.

## Supplementary Information

Supplementary Information 1.

Supplementary Information 2.

Supplementary Table 1.

Supplementary Table 2.

Supplementary Table 3.

## Data Availability

All data are available in this manuscript.
